# Application of a Multi-Layer Perceptron and Markov Chain Analysis-Based Hybrid Approach for Predicting and Monitoring LULCC Patterns Using Random Forest Classification in Jhelum District, Punjab, Pakistan

**DOI:** 10.3390/s24175648

**Published:** 2024-08-30

**Authors:** Basit Aftab, Zhichao Wang, Shan Wang, Zhongke Feng

**Affiliations:** Precision Forestry Key Laboratory of Beijing, Beijing Forestry University, Beijing 100083, China; basit.raja292@hotmail.com (B.A.); zhichao@bjfu.edu.cn (Z.W.); wangshan@bjfu.edu.cn (S.W.)

**Keywords:** land-use land-cover, Markov chain model, multi-layer perceptron, random forest, sustainable land, remote sensing

## Abstract

Land-use and land-cover change (LULCC) is a critical environmental issue that has significant effects on biodiversity, ecosystem services, and climate change. This study examines the land-use and land-cover (LULC) spatiotemporal dynamics across a three-decade period (1998–2023) in a district area. In order to forecast the LULCC patterns, this study suggests a hybrid strategy that combines the random forest method with multi-layer perceptron (MLP) and Markov chain analysis. To predict the dynamics of LULC changes for the year 2035, a hybrid technique based on multi-layer perceptron and Markov chain model analysis (MLP-MCA) was employed. The area of developed land has increased significantly, while the amount of bare land, vegetation, and forest cover have all decreased. This is because the principal land types have changed due to population growth and economic expansion. This study also discovered that between 1998 and 2023, the built-up area increased by 468 km^2^ as a result of the replacement of natural resources. It is estimated that 25.04% of the study area’s urbanization will increase by 2035. The performance of the model was confirmed with an overall accuracy of 90% and a kappa coefficient of around 0.89. It is important to use advanced predictive models to guide sustainable urban development strategies. The model provides valuable insights for policymakers, land managers, and researchers to support sustainable land-use planning, conservation efforts, and climate change mitigation strategies.

## 1. Introduction

Land-use and land-cover (LULC) changes in urban areas, forest cover, and other vegetation are indicators of human activities [1]. The LULC change trajectory is categorized worldwide by gains in urban land, agriculture, and declines in forests, these changes are associated with the changes in forest land to agricultural expansion, urban growth, and deforestation. Growth has the potential to create new residential constructions, leading to environmental degradation and urban sprawl [2]. The apparent outcome of such transformations is the mismatch between the quantity of land available to residents of agricultural areas around a city and that open to urban residents [3]. Agricultural land next to cities is critical for ensuring food security and generating revenue for a city’s expanding population [4]. The increase in a city’s ecological footprint jeopardizes the fundamentals of sustainable development [5].

Individuals in Pakistan move from rural to urban areas in search for better amenities, placing a great deal of strain on the country’s finite natural resources [6]. Additionally, due to a lack of thorough planning and management, urbanization frequently expands unsystematically, leading to uncontrolled growth [7]. The LULC pattern changes are significantly impacted by the unchecked and disorderly growth of urban development [8]. Numerous environmental problems may arise as a result of growing urbanization, such as air pollution, water pollution, soil degradation/erosion, and loss of green spaces [9]. Examining the variables that are crucial in causing the overall changes is a significant part of analyzing LULC change categories [10]. Urban centers in Pakistan have grown rapidly in size and number without following the accepted standards for being classified as planned or unplanned [11]. There are not many well-established standards for separating cities into planned and unplanned metropolitan areas. LULCC is primarily driven by factors such as population growth, which spurs urban expansion and increases resource demands [12]. Economic development leads to increased agriculture, mining, and infrastructure projects [13]. Climate change affects weather patterns and vegetation [14]. Other factors include government policies, which can either promote conservation or facilitate further development [15].

Satellite remote sensing and GIS are the most widely used techniques for quantifying, mapping, and detecting LULC patterns due to their precise geo-referencing techniques, digital formats appropriate for computer processing, and repetitive data acquisition [16]. Based on multi-temporal remotely sensed data, the digital change detection process has been widely used to determine and describe the prevalent LULC change properties. The data’s ability to detect unusual changes between two or more dates is the primary use case for change detection. The issue of precise LULCC monitoring in a variety of settings has been studied by a number of researchers [17]. Numerous studies have addressed the land-use and land-cover changes in arid and semi-arid regions that are productive for agriculture. Recent studies have provided valuable insights into the dynamics of land-use and land-cover changes (LULCCs) across diverse regions. In the Amazon Rainforest [18], researchers highlighted a concerning acceleration in deforestation rates driven by illegal logging and agricultural expansion, as revealed through high-resolution satellite imagery. In Jakarta [19], researchers documented the rapid impact of urban sprawl on land cover and water resources, emphasizing the challenges posed by expanding urban areas. Ref. [20] focused on the Mississippi River Delta, revealing continued wetland loss despite restoration efforts, largely due to subsidence and sea-level rise.

The district area has been constantly under pressure from population growth, urbanization, and faster economic growth. Between 1990 and 2015, there was a notable decrease in surface water bodies and a significant increase in impermeable cover within the city limits, according to recent research [21]. Expert analysis [22] predicts that the Jhelum district will change dramatically by 2035, transforming from a green city to a concrete metropolis. But the city’s resources and infrastructure are not ready to handle the problems brought on by the rapidly growing population. According to official data, the population of the Jhelum district has increased by 100% in the previous 20 years, with 3.5 million people living there as of the 2019 census [23]. Unfortunately, this condition was made worse by the district’s susceptibility to both natural and man-made environmental changes. The most urgent issue was LULC changes because urban growth has resulted from the unchecked growth of housing societies and roads. The government of Punjab had declared approximately 630 housing societies to be unlawful. Consequently, a thorough analysis of the dynamics of the extended LULC transformation is necessary, as this is essential to understanding and evaluating the various environmental changes. According to [24], this kind of examination makes it easier to achieve sustainable development goals and lessen the impact of environmental changes on the planet.

Researchers from all over the world have used a variety of approaches to predict future LULC changes in their fields of study. A new approach called the MLP-MCA-based hybrid approach has been used to combine the multi-layer perception (MLP) model and the Markov chain analysis (MCA) model. By combining the advantages of both multi-layer perceptron and Markov chain analysis, the proposed novel hybrid approach seeks to overcome the shortcomings of each technique independently and more successfully handle particular problems and tasks than the current approaches. The scientific community has shown a great deal of interest in this integrated technique because it can increase the accuracy of predictions and improve the overall performance of models [25]. When simulating LULC changes, the MLP is an effective tool used to generalize transition potentials. The supervised backpropagation algorithm, which enables the MLP model to be practically trained, is used to accomplish this [26]. The probability of changing states over time is analyzed by the statistical model known as the mean change analysis (MCA). MCA forecasts land-use LULC changes over a specified time frame. In particular, the model uses historical data to estimate the probability of changing from one land-cover class to another [27]. However, by combining the best features of the two different MLP and MCA approaches, the MLP-MCA hybrid methodology creates a more accurate forecast of future developments and a thorough map of urban expansion [28].

Different methods for predicting spatial changes are available in current land-use prediction models, each with advantages and disadvantages [29]. The system dynamics model—while it may lack spatial details—is excellent at capturing long-term trends and feedback loops [30]. Cellular automata models can be computationally demanding and may depend on arbitrary rules, but they are effective at simulating spatial interactions and local dynamics [31]. While Markov models offer a simple, probabilistic method for predicting transitions; they frequently ignore intricate spatial connections. The CLUE-S model combines geographical characteristics with land-use demands, providing precise regional insights but necessitating a significant amount of calibration [32]. Agent-based interactions are incorporated into the FLUS model, which increases flexibility but requires extensive behavioral data [33]. The PLUS model combines spatial and temporal dynamics, which can be complex to design and validate [34]. In contrast, the proposed MLP-MCA (multi-layer perceptron with multi-criteria analysis) method leverages advanced neural networks to model intricate, non-linear relationships in land-use data and it integrates multiple decision criteria, offering enhanced accuracy and adaptability. MLP-MCA, despite its need for large datasets and complex tuning, provides promising results by combining machine learning with comprehensive decision-making frameworks, addressing many limitations of traditional models [35].

The study area has exhibited a notable urban expansion trend [36], leading to the congestion of previously developed regions over time. There is limited research on past changes in the study area despite the obvious significance of LULC transformations [37]. However, no research has been conducted before to predict and assess the LULC changes using the MLP-MCA method in the Jhelum district. The objective of this study is to identify changes in land-use and land-cover (LULC) patterns, gains, losses, and spatial trends from 1998 to 2023. We also used a hybrid approach based on multi-layer perceptron and Markov chain analysis to predict the dynamics of LULC changes in Jhelum by 2035, providing insightful information about the current state of urban growth and future expansion potential; the study’s findings will be very important to urban planners and policymakers.

This study focuses on land-use and land-cover changes (LULCCs) by leveraging multi-layer perceptron (MLP) and Markov chain analysis (MCA) to enhance the accuracy of monitoring and prediction. The rationale behind utilizing this methodology was its capacity to capture intricate, non-linear correlations in LULCC data, thereby furnishing more accurate and practical insights for land management and policy formulation. The 1998–2023 timeframe was selected because it encompasses significant environmental and socioeconomic changes relevant to the research topic. The availability of high-resolution satellite images and remote sensing data, which are essential for an in-depth study, is guaranteed by this timeframe. Additionally, by selecting this period of time, it was feasible to assess the immediate and long-term impacts of past and present land management intervention patterns and policy modifications, providing a thorough understanding of land-cover dynamics and their implications for sustainable development.

## 2. Materials and Methodology

### 2.1. Study Area

**Figure 1:**
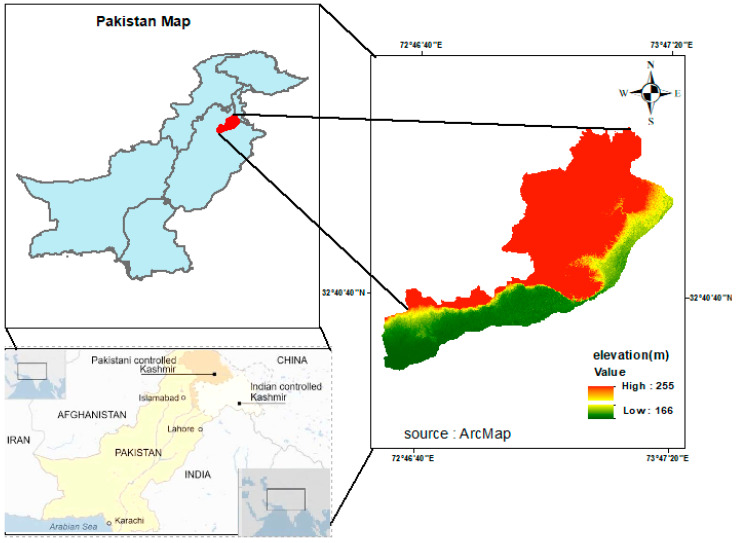
Geographical location of the study area.

The Jhelum district is located between the geographical coordinates of 32.9337° N latitude and 73.7279° E longitude, as shown in Figure 1. Jhelum is one of the populated districts located in the province of Punjab, Pakistan. The Jhelum district has a population of approximately 1.2 million people and experiences a subtropical climate with moderate to high annual rainfall [38]. The average annual precipitation is about 800 to 1000 mm and the HDI is about 0.741 [39]. The district topography is characterized by a mix of plains, hills, and rivers, and has a diverse topography, ranging from the flat plains of the plateau to the hilly areas of the salt range. The Jhelum River is one of the five rivers of Punjab that flows through the district. The district has experienced infrastructure developments throughout the years, including the construction of roads, bridges, and public buildings [40,41]. Efforts have been made to improve the overall transportation network, and there has been some development in the health and education sectors. Jhelum has a semi-arid climate, with hot summers and relatively cool winters. The summer season, from May to September, is hot and dry, with temperatures often rising above 40 °C. The winter season, from November to March, is relatively mild, with temperatures occasionally dropping to around 5 °C.

### 2.2. Data Acquisition

**Figure 2:**
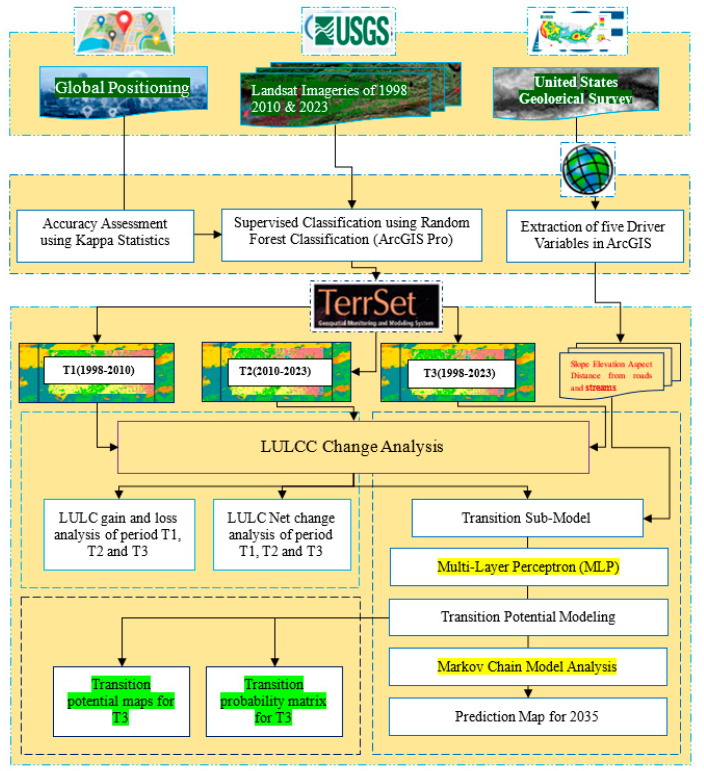
Methodology flowchart.

To achieve this study’s primary objective, we required digital elevation models, field data, and vital spatial data such as Landsat imagery. We obtained the digital elevation model (DEM) and Landsat imagery from the United States Geological Survey (USGS) for the years 1998, 2010, and 2023. In order to determine the slope, aspect, elevation, distance to roads, and distance to streams, among other terrain-related parameters, this study used a high-resolution digital elevation model (DEM) dataset with a spatial resolution of thirty meters as its major data source. Landsat 5 and 8 imagery were collected for the years 1998, 2010, and 2023 to study the LULC changes. In order to mitigate the impact of clouds and seasonal variations on reflectance values, image acquisitions were conducted in March, coinciding with the dry season in the Jhelum district study area. Table 1 presents a comprehensive summary of the satellite data that were used in the research, outlining relevant information and specifications. The investigation’s technique is illustrated in the Figure 2 schematic diagram, which provides a concise and detailed summary of the method’s processes.

### 2.3. Image Pre-Processing

To establish a more direct relationship between the acquired data and biophysical phenomena, it is crucial to pre-process satellite images prior to change detection phenomena [42]. Remote sensing data that were obtained from aircraft or satellites were typically geometrically distorted due to the movements of the acquisition system and platform. For geometric correction, the satellite data were loaded into the ENVI 10.3 program in image format. Following geo-referencing, the photos were mosaicked, and the area of interest (AOI) was subsetted. As part of the analysis of all the satellite data, per-pixel signatures were assigned and the land area was divided into five classes based on the distinct digital numbers (DNs) of different landscape characteristics. The categorized classes were as follows: vegetation, built-up area, forest land, waterbody, and barren terrain. (Table 2). To set the classes apart, each class was given a distinct identity and a specific color. By defining polygons around representative sites, training samples were chosen for each predetermined land-cover and land-use type. The spectral signatures of the different land-cover types obtained from the satellite imagery were recorded in the pixels encircled by these polygons. As stated [43], a satisfactory spectral signature guarantees “minimum confusion” among the land-cover types that need to be mapped. To extract the desired thematic information from the Landsat images, an automated classification process was applied, resulting in the assignment of all pixels to distinct LULC categories [44].

### 2.4. Multi-Layer Perceptron and the Markov Chain Method

The combination of random forest (RF), multi-layer perceptron (MLP), and Markov chain analysis (MCA) provides a potent method for assessing and forecasting land-use and land-cover (LULC) changes [45]. RF—with its ability to categorize land-cover categories and evaluate features from remote sensing data—offers a strong basis for comprehending land-cover patterns as they exist now. In order to provide deeper insights into changing land-cover dynamics, MLP improves this by modeling intricate, non-linear interactions and forecasting future changes based on the categorized data. In addition to these techniques, MCA provides the ability to anticipate future land-cover situations based on past patterns by analyzing the probability of transitions between various land-cover states across time [46]. By combining these techniques, researchers can achieve a comprehensive and accurate analysis of land-cover changes and improve decision-making for land management and environmental conservation. This integrated approach leverages the strengths of each method to address the multifaceted nature of LULC, ultimately leading to more precise and actionable insights [47].

The hybrid method known as MLP-MCA (multi-layer perceptron with Markov chain analysis) combines powerful machine learning algorithms with a structured decision-making framework to provide an improved approach to land-use prediction [48]. Fundamentally, the MLP component models intricate, non-linear interactions between land-use variables using a neural network architecture made up of several layers of connected neurons [49]. The model was able to identify complex patterns and interactions within large datasets due to this deep learning technique, which produces predictions that are more precise and nuanced [50]. This was enhanced by the MCA component, which adds more criteria to the decision-making process and assesses a range of variables, including the effects on the economy, the environment, and society. The model can adjust to changing conditions and evolving data thanks to the integration of MLP and MCA, offering a thorough and dynamic study of various land-use situations [51]. 

The MLP-MCA method integrates the strengths of advanced machine learning with probabilistic modeling to enhance land-use forecasting [52]. In order to generate predictions for future land-use scenarios, this method was used with the multi-layer perceptron (MLP) neural network to first evaluate historical land-use data in order to find complex, non-linear patterns and correlations between land-use variables. The Markov chain analysis (MCA) component was used in these predictions to compute transition probabilities based on the MLP outputs [53]. In order to simulate and forecast future land-use changes, MCA makes use of these probabilities, taking into account the stochastic nature of land-use transitions as well as the complex data that are driven by insights offered by the MLP. This hybrid method combines the detailed, adaptive capabilities of MLP with the robust probabilistic framework of MCA, resulting in a more accurate and comprehensive prediction of land-use changes, and effectively addressing the limitations of traditional models [54].

This study evaluated LULC transitions based on LULC maps created by classified Landsat satellite imagery for the years 1998, 2010, and 2023. The Markov land change modeler (LCM) was used to analyze LULC changes and simulate and predict future modifications. This feature-rich framework was available through TerrSat2020 v19.0.8, Geospatial Monitoring and Modeling Software. A wide range of modeling techniques are included in the LCM, which forecasts and analyzes LULC changes and assesses the forecasts in light of previous changes [55]. Multi-layer perceptron, decision forests, support vector machines, logistic regression, sim weight multinomial logistic regression, cellular automata, Markov chain analysis, and artificial neural networks are a few of these techniques. The LCM makes mapping changes, categorizing changes between different LULC categories, and modeling and forecasting future landscape situations easier by incorporating user-specified change factors using IDRISI, 2012. The basic framework used by the LCM to characterize changes was obtained by aligning the LULC maps of earlier and later periods in a Markov chain matrix [56]. In order to assess the Markov chain prediction, a matrix was created to calculate the area of each LULC class for the future time period as well as the rate of change for each LULC transition. The possibility of each transition was calculated in order to assess the change potential [57]. This model is useful for understanding the dynamics of land-use and land-cover (LULC) systems work, as well as for supporting important planning and policy decisions. Moreover, it may predict future LULC changes under various circumstances [58]. LCM is useful for a wide variety of scientific inquiries in a number of studies [59]. The basis for the change analysis involves the variations in LULC between time one and time two [60].

### 2.5. Kappa Statistic Index

The overall accuracy, user accuracy, producer accuracy, and kappa statistics were calculated by the confusion matrix. The kappa coefficient was calculated using Equation (1) [61]. According to the authors of Ref. [62], a kappa coefficient value below 0.4 indicates poor agreement, a value between 0.4 and 0.8 suggests moderate agreement, and a value larger than 0.8 shows strong agreement. In order to assess the accuracy of land-cover maps that were extracted from satellite photos, the various land-cover classes in the region were represented using a stratified random technique. Two hundred samples were used to survey the accuracy based on visual interpretation and ground truth data. An error confusion matrix was used to compare the classification results with the reference data. As it represents diagonal elements and all the components in the confusion matrix, a non-parametric kappa coefficient was also used to quantify the degree of classification accuracy [63].

κ = 1 − Pe/Po − Pe(1)

where

Po is the observed proportion of agreement between raters.
Pe is the proportion of agreement expected by chance.

### 2.6. Prediction and Validation of Land-Use Changes

To predict the LULC changes for the year 2035, TerrSat2020 v19.0.8 (Geospatial Monitoring and Modeling System) software-embedded multi-layer perceptron (MLP) and Markov chain analysis (MCA) were employed. Using Markov chain analysis, the modification amount was forecasted using two different LULC maps (i.e., 1998 and 2023) along with the selected date. Through the use of MLP methods, the exact transition magnitudes were determined and then added to Markov chain probability matrices to aid in the prediction of future LULC changes. Changes to the LULC were predicted using the results of the Markov chain analysis and the sub-model transitions, with the 2023 year as the baseline. The output of the MCA matrix showed the estimated amount of change for each evaluated step leading up to the target completion date. The transition probability matrix for the years 1998–2010 was calculated in order to predict the LULC map for 2023. To validate the model, the expected and observed maps were usually compared. Thus, in order to obtain the predicted and observed LULC map from 2023, the model was validated using kappa index statistics [64]. Afterward, MLP-MCA forecasting for 2035 was performed.

## 3. Results

### 3.1. LULCC Classification Scheme

**Figure 3:**
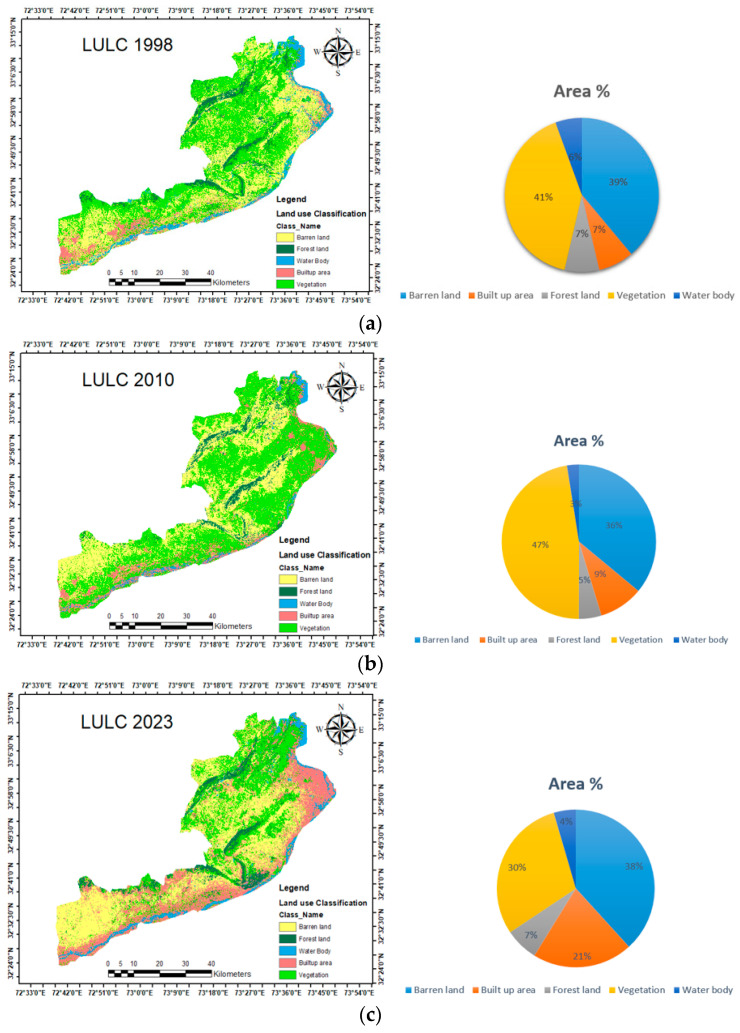
(**a**) Classified land-cover map of the Jhelum district for the year 1998. (**b**) Classified land-cover map of the Jhelum district for the year 2010. (**c**) Classified land-cover map of the Jhelum district for the year 2023.

After the image pre-processing step, LULC classification was carried out in the ArcGIS Pro environment using a supervised classification method that used the random forest (RF) algorithm. The LULC classification process was carried out using the random forest (RF) method in the ArcGIS Pro 2.9 software environment. As a supervised approach based on decision trees and improved bagging and bootstrap techniques, the random forest classifier was suggested. The RF algorithm, which is a part of the supervised classification approach, is now one of the most helpful classifications utilized for Landsat imagery [65]. ArcGIS Pro software was utilized for mapping and multi-temporal image classification. The classified images were verified, validated, and classified using the training data. The spatial and statistical distributions of LULC classes for the years 1998, 2010, and 2023 are presented in Figure 3a–c and Table 3. The study revealed that overall classification accuracy rates for Landsat-classified images for the years 1998, 2010, and 2023 were 94.93%, 95.17%, and 95.23%, respectively. Additionally, 89.84%, 92.43%, and 91.67% of the kappa coefficients were determined for the classified images for the years 1998, 2010, and 2023, respectively.

### 3.2. Accuracy Assessment of LULCC

LULC maps created using supervised classification usually contain some errors; thus, the results of the classification accuracy needed to be assessed. The associated categorized images were evaluated according to how well they depict ground reality when determining accuracy. Classification accuracy must be evaluated to determine trust in the outcomes and the subsequent change detection [66]. To evaluate the accuracy, the classified LULC maps were compared to ground truth data (GPS points). The most widely used and effective technique for gauging the accuracy of classified imagery generated from remotely sensed data is an error/confusion matrix [67]. Based on ground truth data and visual interpretation, the accuracy was surveyed using 250 points and these points were imported into a classified map before being cross-checked. Ground reference data were obtained through the use of stratified random sampling. Every plot had a resolution measuring 30 by 30 m. Based on the information gathered in the field, a LULCC class identification number was assigned to each plot. The classifiers were trained using roughly 70% of the reference data, with the remaining 30% utilized from quick birds to evaluate the accuracy assessment. Several vector and grid data types, including roadways, population centers, and DEM 30 m, were utilized to create slopes, aspects, and elevations to create the vector map layers. Using confusion matrices, and incorporating commission and omission errors, overall accuracy, and the kappa value, classification accuracy processes were established.

The overall accuracy and kappa statistics for the land-cover images for the years 1998, 2010, and 2023 are shown in Table 4.

### 3.3. Land-Use Change Analysis

The basis for the change analysis involves variations in LULC between time one and time two [68]. LULC change analysis is used to determine a transition from one LULC category to another. This study employed contingency table analysis to measure LULC changes over three different time periods: 1998–2010 (period 1), 2010–2023 (period 2), and 1998–2023 (period 3). The findings of this study made it possible to map, identify, and measure the areas where particular LULC classes changed over time [69]. The study investigated the LULC changes over time by monitoring the fluctuations in the gains and losses of LULC classes, the associated impacts on the net changes in a built-up area, and a spatial trend of changes in a built-up area during the relevant periods of interest (periods 1, 2, and 3). A thorough description of the LULC changes from 1998 to 2010, from 2010 to 2023, and from 1998 to 2023 is presented in Table 5, respectively. The study area has undergone significant changes in the last few years, especially in regions classified as built-up areas, forest land, vegetation, and barren land. According to the findings, the built-up area amount increased from 266.75 km^2^ (7.45%) in 1998 to 331.57 km^2^ (9.26%) in 2010, and then to 734.58 km^2^ (20.53%) by 2023. The built-up area increased by 1.81% between 1998 and 2010 and by 11.27% between 2010 and 2023, with a higher and more notable growth rate of 17.57% between 1998 and 2023. The built-up area had a notable shift, growing by 20.53% between 1998 and 2023. The overall rise in the built-up area throughout the study period was facilitated by the significant loss of vegetation (10.92%), barren land (3.02%), and forest land (0.53%) that resulted from this development. According to [70], the built-up area in the Jhelum district increased from 6.9% (210 km^2^) to 17.89% (604 km^2^) between 1990 and 2018. The population expansion and increasing demand for land and urban resources were the main causes of the built-up area’s dynamic growth. A thorough description of the LULC changes from 1998 to 2010, from 2010 to 2023, and from 1998 to 2023 are presented in Table 5, respectively.

### 3.4. Selection of Map Change Transitions

There are minor to major shifts between the LULC maps of the two time periods. In terms of the study objectives, only noteworthy changes between LULC classes were taken into account. The dynamics of the research region were significantly affected when only effective transitions were included. Critical transitions were added to the transition sub-model in order to enhance the multi-layer perceptron neural network’s performance [71]. Seven major shifts in the Jhelum district that were assumed to be potential causes of LULC changes were taken into account. We considered the following transitions: vegetation to forest land, vegetation to barren land, vegetation to built-up area, and forest land to vegetation.

### 3.5. Sub-Structure Model and Variables Selection

It is not possible to generalize the causes that influence land-use and land-cover changes; instead, each research region’s particular elements must be taken into account. However, as many scholars have noted, the applicability changes depending on the circumstances [72]. We refer to the elements that impede the growth of built-up areas as constraints. Among many other physical restrictions, these include the already-existing built-up area, rivers, and road systems. In this study, road networks were taken into account as constraints. When all of the transformations were combined into a collection of sub-models, the potential explanatory power of the variables was examined. The model includes both the dynamic and static components of these variables [73]. For this study, one dynamic and four static variables were taken into account. This study classified the distance from the main roadways as a dynamic variable, while factors like slope, aspect, elevation, and distance from streams were deemed static variables. Static variables were necessary for the transition under discussion because they did not change over time.

**Figure 4:**
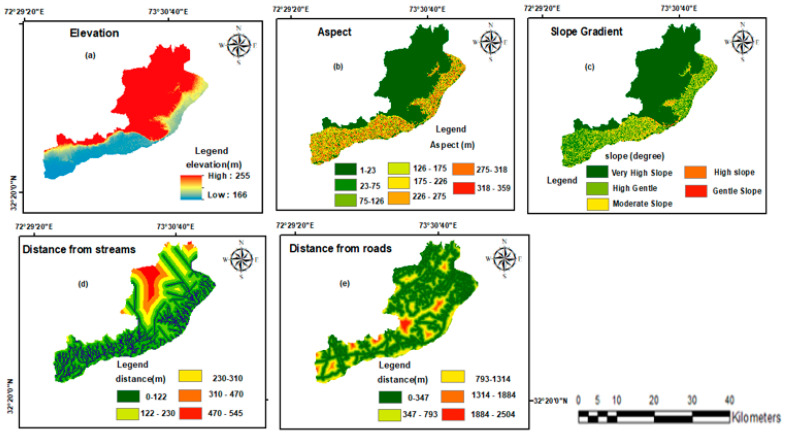
(**a**) Elevation, (**b**) aspect, (**c**) slope gradient, (**d**) distance from streams, and (**e**) distance from roads.

Dynamic variables were calculated continually during a forecast and varied over time. The choice of the dynamic variable “distance to major roads” was made in light of the development and construction activities that occurred alongside roadways. The primary driving factors and LULC transitions are two model variables that have already been established. LULC transitions were further subdivided into sub-models, contributing to the primary driving forces using IDRISI, 2010. Different variables specific to each sub-model were employed to account for specific LULC transitions during the specified time frame. In order to visualize the LULC class’s readiness for LULC future scenarios, transition possibility maps were created. Figure 4 displays the factors utilized in this study.

### 3.6. Modeling Transition Potential Changes

Using the transition potentials tool, significant LULC transitions were found, and in order to efficiently run the primary model under consideration, precise transition potential images were created. To extract the samples for this study, two LULC maps from 1998 and 2023 were utilized. After 10,000 iterations, the MLP algorithm showed that between 1998 and 2023, at least 67,312 cells changed from one LULC category to another. Half of the cells were chosen for training throughout this process, while the other half were given the validation task. A dataset with seven different LULC classes and seven transition classes—in which individual cells changed their LULC status over a specified period of time—was used to train the neural network. After the MLP algorithm was finished, the calibration was measured with an accuracy rate of 90.32%, indicating precise outcomes. The transition maps produced by this study were then used to forecast LULC changes at later times.

### 3.7. LULC Maps and Accuracy Assessment

LULC maps were created using Landsat imagery and the random forest classification algorithm for the years 1998, 2010, and 2023. The spatial, quantitative, and graphical distributions of LULC classes during the periods of three distinct years, 1998, 2010, and 2023, are vividly illustrated in Figure 3 and Table 3. The confusion matrix was used to obtain producer and user accuracy results as well as kappa statistics and total classification accuracy values, which are summarized in Table 4. The study found that the overall classification accuracy rates of the classified images for the years 1998, 2010, and 2023 were 89.26%, 91.72%, and 90.23%, respectively. Additionally, kappa coefficient values of 89.84%, 92.43%, and 91.67% for the classified images from 1998, 2010, and 2023 were achieved. Analyses of LULC class variations throughout different time periods were conducted: period T1 (1998–2010), period T2 (2010–2023), and period T3 (1998–2023). The LULC classifications showed significant changes in the last few years, especially in the areas classified as built-up areas, forest land, vegetation, and barren land. The number of built-up regions increased, according to the findings, from 266.75 km^2^ (7.45%) in 1998 to 331.57 km^2^ (9.26%) in 2010, and then to 734.58 km^2^ (20.53%) by 2023.

The built-up area increased by 1.81% between 1998 and 2010 and 11.27% between 2010 and 2023, with a higher and more notable growth rate of 17.57% between 1998 and 2023. The region’s forest land has gradually decreased over the past 25 years, going from 260.75 km^2^ (7.27%) of the total area in 1998 to 170.17 km^2^ or 4.75%, in 2018, with an overall decrease from 266.75 to 244.77 km^2^ between 1998 and 2023. In 1998, the area covered by vegetation was 1458.45 km^2^ (40.76%) of the total area. Further, it decreased to 1067.7 km^2^ (29.84%) of the total area in 2023. In general, there was a slight increase in the vegetative area. The amount of bare land was 1392.71 km^2^ in 1998, (38.91%) of the total land area. In 2010, the area decreased to 1288.79 km^2^, indicating a 4.65% dropdown. In 2022, the area of bare land increased to 1368.39 km^2^ (38.25%)of the total land area. The waterbody also decreased between 1998 and 2023, from 199.64 km^2^ (5.57%) to 161.44 km^2^ (4.51%).

Throughout the entire period of 1998–2023, the area of barren land experienced a modest decrease from 1392.71 km^2^ (38.91%) to 1368.39 km^2^ (38.25%), representing a loss of 1.23%. Similarly, the vegetation area also decreased from 1458.45 km^2^ to 1067.7 during this period, with a loss of 15.3%. In 1998, forest land coverage accounted for 260.37 km^2^ (7.27%) of the total area during period 3, however, by 2023, it had dropped to 161.44 km^2^ (4.51%) of the entire area, meaning that 4.25% had been lost in that time. Table 3 illustrates the considerable rise in the built-up area, which goes from 266.75 km^2^ (7.45%) of the total area in 1998 to 734.58.74 km^2^ (20.53%) in 2023.

### 3.8. Land-Use Change Gain and Loss Analysis

**Figure 5:**
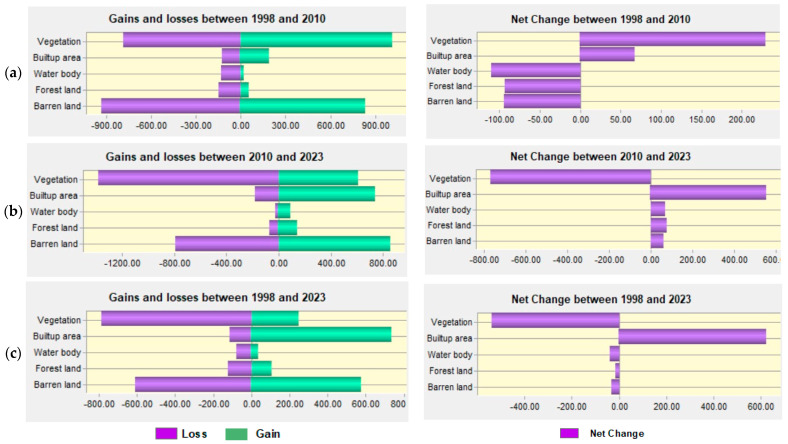
Gains and losses in LULC (sq. km), net change (sq. km) during (**a**) 1998–2010, (**b**) 2010–2023, and (**c**) 1998–2023.

Land-use and land-cover maps from 1998, 2010, and 2023 were utilized to examine the LULCC changes over three different time periods. Most of these classes show both positive and negative changes as shown in Figure 5.

During period 1 (1998–2010), the LULC changes were quantified for various LULC classes. In particular, there was a 60.26% rise and a 46.30% decrease in the vegetated land, with a net loss of 14.04%. There was a 61.31% drop in barren land and a 63.11% increment in it, with a net gain of 2.21%. There was a net gain of 28.74% from the 53.85% decrease and 82.59% rise in the built-up area. Furthermore, a 40.43% decrease in forest land and a 58.64% increase were observed in the following years, yielding a net gain of 18.21%. Finally, the waterbody experienced a decrease of 26.56% and an increase of 58.72%, yielding a 32.16% net gain.

In period 2 (2010–2023), the vegetation land experienced an 82.20% reduction and a subsequent 67.22% increase, resulting in a net gain of 2%. Bare land showed a decline of 88.60% and an increase of 85.91%, with a net loss of 2.69%. There was a 14.28% increase and a 1.84% decrease in the built-up area, yielding a 12.44% net gain. Forest showed a decline of 12.79% and an increase of 9.77%, with a 3.02% net loss. Lastly, waterbodies experienced a 12.68% reduction and a subsequent 4.75% increase, resulting in a net loss of 7.93%.

Regarding overall changes from 1998 to 2023 (period 3), vegetation land experienced a 54.15% reduction and a subsequent 27.13% increase, resulting in a net loss of 27.02%. A 44.04% decline in barren land was followed by a 42.74% rise, for a net loss of 1.30%. There was an 82.48% increase and a 41.85% decrease in the built-up area, yielding a 40.63% net gain. There was a net loss of 3.66% due to the 45.91% decrease and 42.25% increase in forest land. Finally, waterbodies experienced a 39.07% decrease followed by a 42.74% increase, for a net loss of 3.67%.

### 3.9. Spatial Trend Analysis of LULC Maps

**Figure 6:**
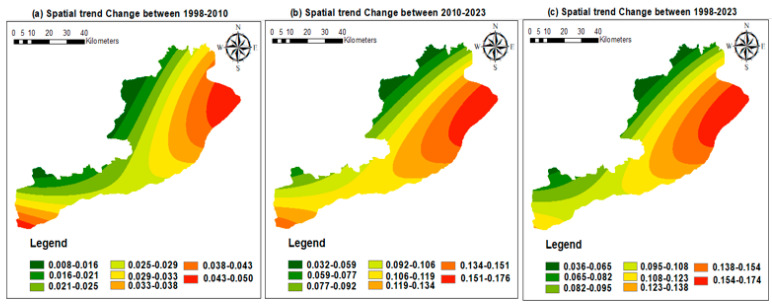
Built-up area spatial trends of change from the years (**a**) 1998 to 2010, (**b**) 2010 to 2023, and (**c**) 1998 to 2023.

A useful method for visualizing and providing a generalized pattern of changes is to analyze the spatial trends in LULC changes, which is based on analyzing two observed land cover maps. Throughout three distinct periods, 1, 2, and 3, the study created maps showing the patterns of transitions from all LULC classes to built-up areas using a spatial trend analysis tool. The trend maps were made using the default third-order polynomial parameter, which was determined to be the best fit for the observed trend of change. Figure 6 illustrates the spatial tendencies of the LULC maps. Lower and higher values, corresponding to fewer or larger changes, showed the amount and direction of the changes. In comparison to other regions, the central section of the research area showed a more marked transition of built-up areas, suggesting a notable increase of built-up areas in a westward direction.

### 3.10. Transition Probability

The transition probability matrix used to project the LULC trends for 2023 was computed using the Markov chain approach, based on the LULC maps for the years 1998 through 2010. The produced matrix is shown in Table 6. The expected probability or degree of change from one specific LULC classification to another throughout the given duration is represented by the transition probability matrix, which is the result of the contingency tabulation of the LULC images between 1998 and 2010. The transition probability matrix’s rows and columns represent the various LULC classifications that were seen in the images taken at different times. The outcomes of the Markov chain analysis provide valuable and important information about the dynamics, patterns, and magnitudes of LULC transformations across time within a certain geographic area. By analyzing LULC maps from two different periods, this stochastic approach produces a conditional probability image and a transition probability matrix.

### 3.11. Prediction and Validation Analysis

The MLP-MCA model is the most effective method for predicting future spatiotemporal changes, especially in high-precision LULC conversions. Using an MLP to model the time series data and a Markov chain to forecast future states based on the MLP output, MLP-MCA combines these two methods. In particular, the MLP method was utilized to generate possible transition maps for multiple LULC transitions with an astounding accuracy of 90.14%.

**Figure 7:**
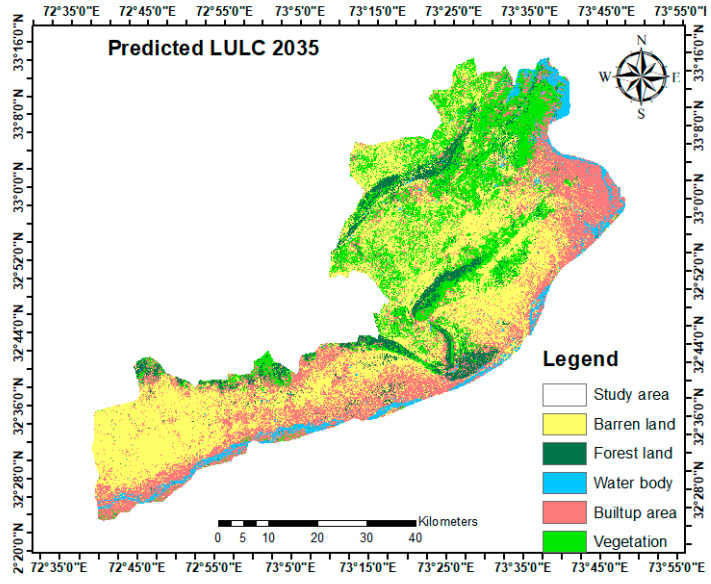
Predicted land-cover map for 2035.

Through a comparative analysis of the projected and actual LULC of 2023, the kappa statistic values for both the location and amount were determined. According to the examination of the kappa statistics study, Kno is 0:8820, Klocation is 0:9021, KlocationStrata is 0:8625, and Kstandard is 0:8813. It has been observed that every kappa index value is more than 0.80. This demonstrates a reputable degree of alignment between the projected and actual LULC. This outcome showed that the MLP-MCA-based hybrid approach was accurate and successful at predicting future LULC trends [74]. The LULC changes in the map for the year 2035 were forecasted after the incredibly successful prediction of the LULC map for 2023. The LULC transition probability matrix and map spanned the period from 2023 to 2035. LULC maps from 1998 and 2023 were used as base maps to predict the LULC transition for the year 2035., as shown in Figure 7. Table 7 provides relevant area statistics about the various anticipated LULC changes for 2035. The Markov chain model feature can be employed to simulate the temporal dependencies among the LULC since the training process elaborated the state transition probabilities from one LULC class to another. This can aid in improving the accuracy of projections regarding land-use and land-cover patterns in the future [75].

According to the MLP-MCA-based hybrid approach prediction, the research area indicates a concerning trend of ecological degradation for the year 2035. In particular, from 2023 to 2035, it was anticipated that the overall area of the waterbody would rise from 161.44 km^2^ (4.51%) in 2023 to 229.55 km^2^ (6.41%) in 2035, representing a gain of 1.29%. The percentage of the total area that will be covered by forest land is predicted to drop from 244.77 km^2^ (6.84%) in 2023 to 220.98 (6.17%) in 2035, representing a loss of 0.67% throughout this time. Additionally, it is anticipated that the area covered by barren land will decline from 1368.39 km^2^ (38.25%) in 2023 to 1297.89 km^2^ (36.28%) in 2035, indicating a loss of 1.97% during this time. Similarly, the vegetation area is expected to decrease from 1067.70 km^2^ (29.84%) in 2023 to 932.64 km^2^ (26.07%) in 2035 of the total area, resulting in a loss of 3.77% during this period. In contrast, the built-up area is projected to increase from 734.58 km^2^ (20.53%) in 2023 to 896.76 km^2^ (25.07%) in 2035 of the total area, indicating a gain of 5.02% during this period in Jhelum, Punjab, Pakistan.

**Figure 8:**
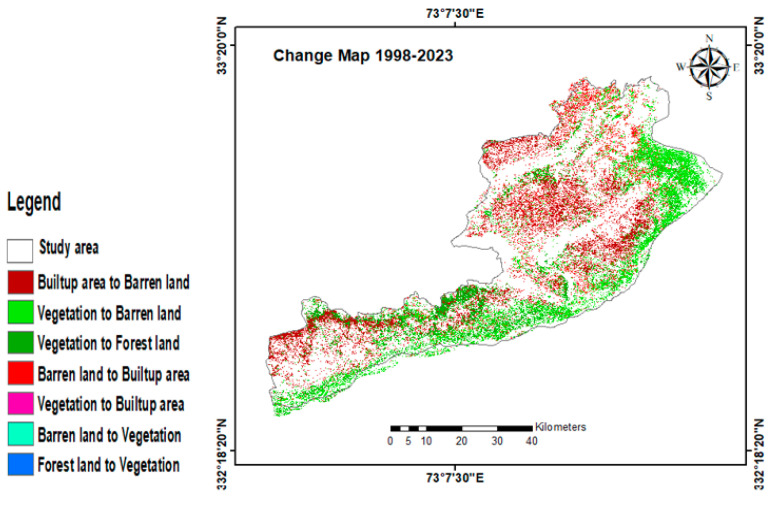
Combined LULC change map from 1998 to 2023.

**Figure 9:**
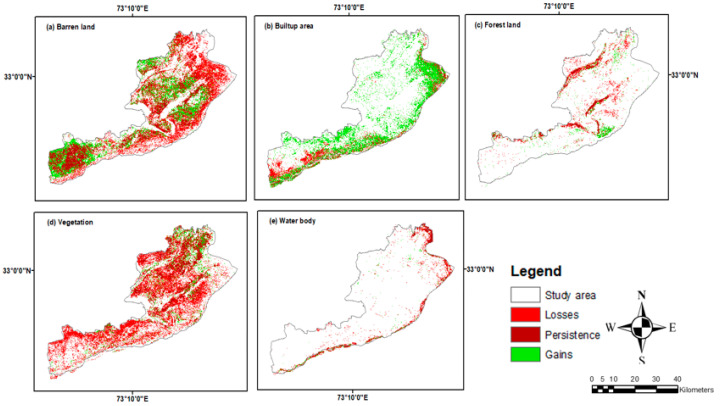
Gains and losses in each class between 1998 and 2023.

The investigation into LULC changes across the years 1998–2010, 2010–2023, and 1998–2023 demonstrates that during all three research periods (periods 1, 2, and 3), there were notable shifts and transitions among the different LULC classes. The percentages of gains and losses in LULC for periods 1, 2, and 3 are shown in Figure 5 together with their respective contributions to the net changes in built-up areas. Moreover, the combined LULC change map from 1998 to 2023 is shown in Figure 8. Finally, the gains and losses in various LULC classes from 1998 to 2023 are shown in Figure 9.

**Figure 10:**
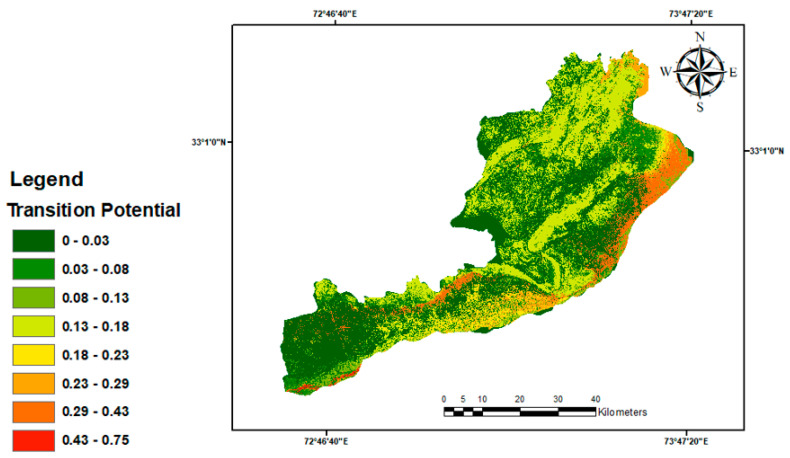
Markov conditional probability map for 2035.

There was a concerning imbalance in the probability of change in the Jhelum district, which indicates a sharp decline in forest land, bare land, and vegetation and a sharp rise in the built-up area. The likelihood of land transitioning from vegetation to built-up areas is expected to increase, ranging from 6.17% in 2023 to 15.45% in 2035. This study suggests that as a result of urban growth or infrastructure development, a greater percentage of vegetative space will likely be converted to built-up areas. On the other hand, the likelihood of a place transitioning from undeveloped to developed territory is expected to increase from 20.9% in 2023 to 29.76% in 2035. This implies that once low-value ecological regions are being converted into metropolitan areas. Nonetheless, there has also been a minor increment in the probability of the shift from forest areas to built-up areas, expected to rise from 2.45% in 2023 to 4.03% in 2035. Even with a small increase, it might still have an impact on biodiversity, ecological services, and the sequestration of carbon that forests offer. Table 6 and Table 8 provide the Markov transition probability matrixes, showing the likelihood of LULC transitions between categories for the years 2023 and 2035. Figure 10 displays the Markov conditional probability map for 2035.

As shown in Table 7 and Figure 7, it is additionally significant that the built-up area is expected to continue to rise significantly (by 7.52% by 2035), resulting in a large loss of vegetation (3.77%), barren land (2.97%), and forest (0.89%). Unfortunately, the alterations to vegetation, forest, and barren land will be accompanied by an unregulated surge in the built-up area. Through the integration of in situ data sources and remote sensing images, we were able to successfully perform a quantitative evaluation of the geographical effects of environmental, social, and economic aspects of LULCC changes in this study. As a result, we obtained a thorough grasp of how urbanization affects the forest, vegetation, and arid land changes in the Jhelum district, Punjab, Pakistan, as well as ecological policies related to these changes. The research determined LULC change patterns from 1998 to 2023 in order to forecast the dynamics of LULC changes in the Jhelum district, Punjab, Pakistan, for the year 2035. Multispectral Landsat satellite images from 1998, 2010, and 2023 were used to perform LULC classification using a random forest classification algorithm in order to accomplish the desired result. The time series data were modeled using a hybrid approach that combined multi-layer perceptron and Markov chain analysis to detect LULC changing patterns, gains, and losses, as well as spatial trends of changes from 1998 to 2023, and to predict future dynamics of LULC changes for the year 2035. The kappa statistics index was used to assess the accuracy of the anticipated LULC map for 2023. Then, to predict potential LULC transformation scenarios for 2035, the MLP-MCA-based hybrid approach was utilized. The forecasted results provide an understanding of both the quantitative and spatial aspects of the anticipated changes, including their possible sizes and distributions. The estimated results for 2035 indicate a notable expansion of 163.16 km^2^ in the built-up area. Simultaneously, 1.79 km^2^ is expected for the waterbody. In comparison, significant reductions of 137.42 km^2^ in vegetation, 23.21 km^2^ in forests, and 172.09 km^2^ in barren areas are predicted. These modifications are expected to take place between 2023 and 2035. The projected substantial increase of 6.46% in the built-up area by 2035 will be especially noteworthy, as it is expected to cause a significant loss in vegetation (3.77%), barren land (1.97%), and forest (0.89%). A significant increase in the built-up area was observed in the LULC change analysis performed from 1998 to 2023. 

## 4. Discussion

Several studies have adopted the use of machine learning algorithms to forecast future LULC trends. In this work, LULC changes were assessed throughout three time periods (1998, 2010, and 2023) using ArcGIS Pro. In addition, the LULC changes in 2035 were predicted using the multi-layer perceptron–Markov model. Three different time periods were used to consistently reach over 86% precision. As a result, all producer groups were classified with an accuracy rate of more than 85%, according to the findings [76]. The validity of the supervised classification method used was supported by our research, which has shown consistently excellent accuracy. The multispectral scanner (MSS) data provided the lowest level of accuracy of 85.6% across all metrics tested [77]. The accuracy and kappa coefficient values of every image from 1986 to 2015 were greater than 75% [78]. In the Jhelum district study, the kappa coefficients showed values of more than 0.85% in each of the four time periods (1998, 2010, and 2023). Ref. [79], which looked at the kappa coefficients exceeding 0.8 for all forest categories, found similar results. Within the research region, LULC changes had a major impact on the distribution of forests, bare land, vegetation, and built-up areas. The majority of land was used for built-up areas, which had increased in quantity. Between 1991 and 2021, Jhelum’s built-up areas increased greatly, while the amount of barren land and vegetation dropped sharply. According to Manna, the Jhelum district built-up area has grown since 1990. For a variety of reasons, including job opportunities, educational pursuits, or entrepreneurial ventures, rural populations have relocated to metropolitan regions [80]. Between 1991 and 2021, a progressive decrease in the amount of vegetation and bare terrain was noted. Additionally, between 1990 and 2020, Ref. [80] discovered a decrease in Islamabad’s agricultural and arid areas, with yearly drop rates of 0.25 and 1.24%, respectively. According to a recent study by Habte et al. (2021), efforts to restore land in Ethiopia’s northeastern region between 1984 and 2005 resulted in a decrease in the amount of bare land. The results of our analysis show that, between 1991 and 2021, the amount of forest area in Jhelum district generally decreased. This current result was consistent with the work by Manna [80]. This study suggests that the hybrid model is the most suitable approach for LULC modeling and prediction, offering enhanced accuracy and valuable insights into land management and planning. 

For the classification of Landsat imagery, the random forest algorithm was used to classify the LULC images by constructing multiple decision trees during the training phase, with each selected on a subset of the data and features. For each tree, a bootstrapped sample of the dataset was used, and at each node, a random subset of features was considered for splitting because it ensured diversity among the trees and reduced the risk of overfitting. When classifying new data, each decision tree in the forest provided a prediction based on its learned patterns, and the final classification was determined by aggregating the predictions from all trees, typically by selecting the most frequent class label. This approach enhances the model’s robustness and accuracy, making it effective for handling complex land-use data and providing valuable insights into feature significance. While global land-use classification results provide a broad overview, due to their limited resolution and generality, they frequently reduce the accuracy and significance required for in-depth study. On the other hand, machine classification techniques offer several benefits due to their ability to adapt to local conditions, which can greatly increase accuracy. These techniques make use of localized, high-resolution data to identify certain environmental characteristics and land-use patterns that may be missed by global datasets. Furthermore, machine classification techniques allow for ongoing updates that take into account recent land-use changes that may not be reflected in global results. Additionally, they use a variety of regional data sources, enhancing the classification’s precision and dependability. All things considered, machine categorization offers more precise, detailed, and contextually relevant land-use data, making it a better method for customized and current analysis.

The Markov chain and MLP neural network approaches in LCM offer insights into the amount and geographical distribution of change in LULC prediction, respectively [81]. This study’s kappa index values all exceed the 80% acceptable cutoff point [82], showing that the simulated and observed LULC maps correlate quite well. A very good area under the curve (AUC) value of 0.88 was also attained [82]. Our analysis indicates that the region is expected to experience a significant increase in urbanization by 2035, with vegetation and bare ground being converted to built-up areas during the study period. The study’s findings show that other land-cover types are being replaced by significant urban growth. By 2035, growth will be expected to cover about 896.10 km^2^ or a net gain of 166.27 km^2^ over land cover in 2023, according to the CA-MC model (Figure 3). Furthermore, the expansion of built-up areas is expected to cause a slight decline in the coverage of forest land. In general, urban expansion is fueled by fast population increase, contributing to the loss of forests. The results of this analysis are consistent with those published by [83], showing that the growth of populated regions is/will be accompanied by a steady decline in other land-use categories, such as forests, both now and in the future. Ref. [84] noted that the built-up area will keep growing as other land uses diminish, according to the CA-MC prediction model. A hybrid Markov chain (MC) and multi-layer perceptron (MLP) modeling approach was utilized to examine the dynamics of LULC changes in Rajshahi, Bangladesh, over the years 2000–2020 and 2020–2040. According to the data, a 17% drop in green cover and a 30% increase in urbanization are expected by 2030.

In this study on LULCC prediction, we employed Markov chain models, random forest methods, and multi-layer perceptron. Satellite remote sensing imagery was an effective way to provide large-scale land-use and land-cover data for improved land-use classification and future prediction (via Landsat data from 1998 to 2023). Moreover, random forest was limited in its ability to represent certain facets of intricate district urban settings. Improving LULC classifications and forecasts of urban growth requires the use of higher resolution and more recent satellite data, particularly from Landsat imagery. Urban expansion estimates that are more accurate may also be obtained by utilizing neural networks and sophisticated deep learning algorithms. Socioeconomic data should be integrated into future research as well. By including land-use policies, economic development, and population expansion in models, urban dynamics can be better understood. Furthermore, examining several scenarios for urban development after 2035 would offer insightful information about sustainable urban planning in the long term.

## 5. Conclusions and Recommendations

The purpose of the study was to investigate the LULCC patterns for the years 1998, 2010, and 2023 in order to predict the future dynamics of LULC changes for 2035 in the Jhelum district. Multispectral Landsat images from the years 1998, 2010, and 2023 were used to perform LULC classification, using a random-forest classification algorithm, in order to accomplish the desired result. To simulate time series data and identify LULC changing patterns, gains, and losses, as well as regional trends of change from 1998 to 2023, a hybrid approach combining multi-layer perceptron and Markov chain analysis was implemented. Future dynamics of LULC changes for the year 2035 were also predicted. The accuracy of the anticipated LULC map for 2023 was assessed using the kappa statistics index. Afterward, possible LULC transition scenarios for 2035 were predicted using the hybrid approach based on MLP-MCA. The study showed that the MLP-MCA-based hybrid approach was one of the best models among the land-use change prediction models in terms of precision, taking into account temporal and spatial factors, combining various data sources, and assisting with policy and decision-making. This indicates that the hybrid model, which offers improved accuracy and insightful information for land management and planning, represents the best method for LULC modeling and prediction. The results showed that socioeconomic circumstances had significantly improved. The urbanization trend resulted in a decline in vegetation, encompassing both forest and arid areas. The simulation’s findings for the anticipated period of 2023–2035 showed a considerable decline in the amount of forest land, bare ground, and vegetation, with reductions of 26.07 km^2^, 36.20 km^2^, and 6.17 km^2^ respectively. A substantial gain of 135.1 km^2^ of the built-up area is expected based on the projected outcomes for 2035, which demonstrate that urgent actions need to be taken. Water bodies are predicted to expand by 68.1 km^2^ at the same time. On the other hand, substantial declines of 23.79 km^2^ in the forest area, 70.5 km^2^ in the barren land, and 123.09 km^2^ in the vegetation area are projected. The findings of this study have the potential to offer policymakers insightful viewpoints into creating effective strategies for future urban land-use planning and management. In addition, this study emphasizes the need for urgent actions to be taken for urban planning and urban forest practices in order to ensure the general preservation of green spaces. Overall, the findings contribute to the broader understanding of LULCC changes in rapidly developing regions and emphasize the importance of incorporating sustainable land management strategies for the long-term well-being of the Jhelum district, Pakistan, and similar regions across the world.

Future research should focus on high-resolution remote sensing imagery, socioeconomic indicators, and climate projections to provide a more comprehensive understanding of land-use dynamics and enhance the model’s accuracy. Advancing the MLP architectures by exploring deeper networks and novel activation functions will better capture complex, non-linear relationships in land-use data. Rigorous calibration and validation techniques, including cross-validation and sensitivity analysis, are essential to ensure the model’s reliability and generalizability. Frequently sharing model data and procedures will encourage cooperation and transparency. The model’s validity and effectiveness in land-use planning will be ensured by including stakeholders in the modeling process and offering tools for decision support. Finally, keeping up with new technologies, like drones and satellite systems, can provide better data and improve land-use analysis.

## Figures and Tables

**Table 1:** Landsat imagery specification.

Year	Acquisition Date	Spatial Resolution (m)	Elevation	Azimuth	Landsat
1998	26 March	30	68.28497482	110.93667828	5
2010	13 March	30	46.5525138	140.20663395	5
2023	24 March	30	31.84888425	154.01356104	8

**Table 2:** Land-use and land-cover categories used in the study area.

LULC Type	Description
Built-up area	All types of developed land are included in the areas, such as commercial, industrial, residential, and metallic highways.
Waterbody	Rivers, streams, canals, lakes, reservoirs, bays, and estuaries are some of the areas.
Forest Land	Deciduous forest land, evergreen forest land, and mixed forest land are among the areas.
Barren Land	Plantations, pastures, groves, vineyards, and nurseries are among the areas.
Vegetation	Areas include forests, grasslands, tundra, deserts, and ice sheets.

**Table 3:** Statistics of LULC changes across different periods.

	1998		2010		2023	
LULC Classes	Area km^2^	%	Area km^2^	%	Area km^2^	%
Barren land	1392.71	38.91	1288.79	36.02	1368.39	38.25
Built-up area	266.75	7.45	331.57	9.26	734.58	20.53
Forest land	260.37	7.27	170.17	4.75	244.77	6.84
Vegetation	1458.45	40.76	1698.15	47.46	1067.7	29.84
Waterbody	199.64	5.57	89.24	2.51	161.44	4.51
Total	3577.92	100	3577.92	100	3577.92	100

**Table 4:** Accuracy assessment of different land-cover maps.

	1998		2010		2023	
	Producer’s	User’s	Producer’s	User’s	Producer’s	User’s
LULC Classes	Accuracy (%)	Accuracy (%)	Accuracy (%)	Accuracy (%)	Accuracy (%)	Accuracy (%)
Barren land	97.86	95.16	97.17	96.43	95.32	94.52
Built-up area	95.51	93.53	89.75	93.24	93.47	92.71
Forest land	86.25	85.28	91.48	89.51	86.28	90.04
Vegetation	88.91	89.80	90.04	92.11	93.01	91.13
Waterbody	92.26	91.84	96.65	94.26	97.31	95.14
Kappa	89.84		92.43		91.67	
coefficient (%)						
Overall	89.26		91.72		90.23	
accuracy (%)						

**Table 5:** LULC changes during 1998–2010 (period 1), 2010–2023 (period 2), and 1998–2023 (period 3).

	1998		2010		Change 1998–2010	
LULC Classes	Area km^2^	Area %	Area km^2^	Area %	Area km^2^	Area %
Barren land	1392.71	38.91	1288.79	36.02	−103.92	−2.89
Built-up area	266.75	7.45	331.57	9.26	64.82	1.81
Forest land	260.37	7.27	170.17	4.75	−90.2	2.52
Vegetation	1458.45	40.76	1698.15	47.46	239.7	6.70
Waterbody	199.64	5.57	89.24	2.51	−110.4	−3.06
	2010		2023		Change 2010–2023	
LULC Classes	Area km^2^	Area %	Area km^2^	Area %	Area km^2^	Area %
Barren land	1288.79	36.02	1368.39	38.25	79.60	2.23
Built-up area	331.57	9.26	734.58	20.53	403.01	11.27
Forest land	170.17	4.75	244.77	6.84	74.60	2.09
Vegetation	1698.15	47.46	1067.7	29.84	−630.45	−17.62
Waterbody	89.24	2.51	161.44	4.51	72.20	2.01
	1998		2023		Change 1998–2023	
LULC Classes	Area km^2^	Area %	Area km^2^	Area %	Area km^2^	Area %
Barren land	1392.71	38.91	1368.39	38.25	−24.32	−1.59
Built-up area	266.75	7.45	734.58	20.53	627.83	17.57
Forest land	260.37	7.27	244.77	6.84	−15.6	0.43
Vegetation	1458.45	40.76	1067.7	29.84	−390.75	−15.45
Waterbody	199.64	5.57	161.44	4.51	−38.20	−1.06

**Table 6:** Markov transition probability matrix for 2023.

LULC Classes	Barren Land	Forest Land	Waterbody	Built-Up Area	Vegetation
Barren land	0.8092	0.0076	0.0065	0.0652	0.0143
Forest land	0.0005	0.8734	0.0098	0.0987	0.0314
Waterbody	0.000	0.0008	0.2065	0.0012	0.0634
Vegetation	0.0065	0.0052	0.0004	0.1423	0.1177
Built-up area	0.0001	0.0002	0.000	0.0310	0.8092

**Table 7:** Area statistics of the projected LULC for the year 2035.

	2023		2035		Change 2023–2035	
LULC Classes	Area km^2^	Area %	Area km^2^	Area %	Area km^2^	Area %
Barren land	1368.39	38.25	1297.89	36.28	−70.5	−1.97
Built-up area	734.58	20.53	896.76	25.07	135.18	4.54
Forest land	244.77	6.84	220.98	6.17	−23.79	−0.67
Vegetation	1067.7	29.84	932.64	26.07	−135.06	−3.77
Waterbody	161.44	4.51	229.55	6.41	68.11	1.9
Total	3577.92	100	3577.92	100		

**Table 8:** Markov transition probability matrix for 2035.

LULC Classes	Barren Land	Forest Land	Water Body	Built-Up Area	Vegetation
Barren land	0.7492	0.0174	0.0049	0.1651	0.0635
Forest land	0.1087	0.7514	0.0021	0.0038	0.1340
Waterbody	0.000	0.0046	0.7909	0.1597	0.0448
Built-up area	0.1636	0.0003	0.0393	0.7632	0.0337
Vegetation	0.1767	0.0239	0.000	0.1029	0.6965

## Data Availability

Land-use land-cover maps for the current study were obtained from https://earthexplorer.usgs.gov/, the United States Geological Survey (USGS), and road maps were obtained from https://www.openstreetmap.org/#map (accessed on 16 July 2024). The DEM model of the study area was also obtained from the United States Geological Survey.
